# Increased neutrophil-to-lymphocyte ratio predicts the development of post-stroke infections in patients with acute ischemic stroke

**DOI:** 10.1186/s12883-020-01914-x

**Published:** 2020-09-01

**Authors:** Lanying He, Jian Wang, Feng Wang, Lili Zhang, Lijuan Zhang, Wang Zhao

**Affiliations:** 1 Department of Neurology, The Second People’s Hospital of Chengdu, Chengdu, 610021 People’s Republic of China; 2 Department of Neurology, The Second Affiliated Hospital of Chengdu College, Nuclear Industry 416 Hospital, Chengdu, 610021 People’s Republic of China; 3 Department of Neurology, Yongchuan Hospital, Chongqing Medical University, Chongqing, China, 610020 People’s Republic of China

**Keywords:** Acute ischemic stroke, Neutrophil-to-lymphocyte ratio, Post stroke infections

## Abstract

**Background:**

Infections could increase the risk of poor outcome in patients with acute ischemic stroke (AIS). The peripheral neutrophil-to-lymphocyte ratio (NLR) is an important indicator of inflammation. The purpose of our study was to investigate the association increased NLR with post stroke infections (PSI) in AIS.

**Methods:**

In this study, we included 606 consecutive patients with AIS within 24 h. The NLR was calculated by dividing absolute neutrophil counts by absolute lymphocyte counts. Receiver operating characteristic (ROC) curve was performed to identify the optimal cut point of NLR for PSI. The relationship between NLR and PSI was analyzed by multivariable analysis.

**Results:**

We assessed 606 consecutive patients with AIS. ROC curve analysis showed that the optimal cut point of NLR for PSI was NLR ≥ 5.79. Compared with no PSI, patients with PSI have higher NLR, older age, higher NIHSS, higher PCT, higher percentage of nasogastric tube feeding and indwelling urinary catheter (*P* < 0.05). Multivariable analysis showed that NLR ≥ 5.79 [adjusted odds ratio (aOR),4.52; 95% confidence interval (CI),3.02–6.76; *P* < 0.001], older age (aOR,1.03; 95% CI, 1.00–1.05; *P* = 0.009), higher admission NIHSS (aOR,1.13; 95%CI, 1.07–1.18; *P* < 0.001), indwelling urinary catheter (aOR1.83; 95%CI, 1.08–3.10; *P* = 0.026], and nasogastric tube feeding (aOR2.52; 95%CI, 1.38–4.59; *P* = 0.003) were associated with increased risk of PSI.

**Conclusions:**

Higher NLR can predict PSI in AIS patients. The NLR may help to select high-risk patients to start intervention in time.

## Background

Infections are the most common complication following acute ischemic stroke (AIS) contributing to unfavorable functional outcome and mortality [1–3]. Post-stroke infection (PSI) included stroke-associated pneumonia (SAP), urinary tract infection (UTI), and other localization’s infection, occurring in up to about 30–40% [4, 5]. Older age, stroke severity, oropharyngeal.

dysphagia, orotracheal intubation are risk factors for PSI [6, 7]. Evidence cumulates that immunomodulation after AIS has been associated with an increased the susceptibility to PSI [8–10].

The neutrophil-to-lymphocyte ratio (NLR) is a commonly used indicator of systemic inflammation and infection. As a reflection of innate and adaptive immune responses, NLR has been widely studied due to its easy obtain from peripheral blood. Increased NLR level with increased neutrophils and decreased lymphocyte, suggesting an imbalanced interaction between stroke-related central and peripheral inflammation. It has been studied as a predictor of bacterial infection, showing better predictive value than traditional markers of inflammation [11–14].

In the last decade, the importance of NLR in acute stroke has aroused the interest of many scholars. Higher NLR may be an independent predictor for poor outcome after AIS [15, 16]. Recent studies had shown that even in AIS patients with thrombolytic or thrombectomy, high NLR increased risks of early neurological deterioration, parenchymal hemorrhage, 3-month poor outcome, and mortality, especially in patients with diabetes [17–20]. A retrospective study also shown that NLR on admission was associated with increased risks of 30-day mortality and 90-day poor outcome in patients with critically ill stroke [21]. The recent study shown that NLR predicted SAP in patients with AIS [22, 23].

At present, there are few studies on the relationship between NLR and PSI. Hence, we assembled a cohort of patients with AIS from three centers to investigate whether elevated NLR value was associated the occurrence of PSI after AIS.

## Methods

### Study population

This study was a multicenter prospective study conducted in three medical centers: The Second People’s Hospital of Chengdu, Yongchuan Hospital of Chongqing Medical University, Nuclear Industry 416 Hospital. The patients with AIS were admitted within 24 h of the onset symptom between March 2014 and December 2017. AIS were evaluated by CT or diffusion-weighted imaging (DWI) using Siemens Magnetom Avanto 1.5 Tesla (Siemens Medical Solutions, Erlangen, Germany). The initial severity of stroke was assessed by National Institutes of Health Stroke Scale (NIHSS). Stroke etiology was classified according to the modified Trial of ORG 10172 in Acute Stroke Treatment (TOAST) criteria in three medical centers.

### Inclusion and exclusion criteria

Patients were enrolled only if they fulfilled the following criteria: 1. Age ≥ 18 years; 2. Admission for first-ever acute ischemic stroke within 24 h; 3. Evidence for ischemic lesions is consistent with clinical presentation; 4. All patients received standard therapy (including antiplatelet, statins, blood pressure control, or intravenous thrombolysis or/and endovascular treatment). The exclusion criteria were the following: 1. Oning infection at admission; 2. a history of previous stroke; 3. Cerebral hemorrhage, hypoxia (arterial oxyhemoglobin saturation < 90%).

### Clinical assessment and definition of PSI

We collected the characteristics and vascular risk factors of the patients. Dysphagia was assessed using non-instrumented swallowing tests at admission. The patients were managed with modifications based on swallowing test. Blood cell counts, lipid, and glucose were obtained within 24 h. The neutrophil count divided by the lymphocyte count yielded the NLR.

Infections included SAP, UTI, and other localization’s infections. Within 7 days of admission, symptoms and signs of infection were monitored, along with key indicators including white blood cell count, procalcitonin (PCT), urine screening, body temperature at least 3 times a day. UTI was diagnosed if the WBC count was elevated and nitrite positive during urine screening, accompanied by leukocytosis, PCT > 50 mg/dl, and urine culture positive.

SAP was diagnosed in according with the modified Centers and Prevention critaria of hospital-acquired, based on clinical and laboratory parameters of respiratory tract infection, and was confirmed by both chest X-ray and CT. Infection can also be diagnosed if the body temperature exceeds 38.0 °C and at least two tests show additional leukocytosis and positive blood culture, but no determined focus. PSI was diagnosed by two experienced neurologists who were blinded to other clinical and laboratory findings during the first 7 days of hospitalization.

### Statistical analysis

First, patients were classified into no PSI and PSI groups. The data are presented as median values (interquartile range [IQR]), numbers (%), or mean values (±standard deviation). To identify differences between subgroups, the Pearson χ^2^ test was used for categorical variables. Distributions of continuous variables were determined by the Kolmogorov–Smirnov test, while Mann–Whitney two sample test was applied in case of non-normal distributions. Second, Receiver operating characteristic (ROC) curve analysis was used to evaluate sensitivity, specificity and to determine the optimal cut point of NLR for PSI. Third, we then performed logistic regressions analyses to determine the association between NLR and PSI, adjusting for all confounders (age, baseline NIHSS score, sex, BMI, hypertension, current smoking, current alcohol drinking, diabetes, hyperlipidemia, thrombolytic therapy, thrombectomy, thrombolytic therapy + thrombectomy, family history of stroke, etiological classification, nasogastric tube feeding, indwelling urinary catheter, and medications use). Results were expressed as adjusted odds ratios (OR) with the corresponding 95% confidence interval (CI). The data were analyzed using SPSS software (SPASS 22.0). *P* values< 0.05 were considered as statistically significant.

## Results

### Characteristics of the study subjects

Six hundred six patients with AIS met the inclusion criteria. The mean age was 66.72 ± 10.35 years (40–96 years), comprised 48.84% (296) men. In the study population, 391 patients had a history of hypertension, 211 had a history of diabetes, 334 had a history of hyperlipidemia, 163 patients current Smoking,183patients current alcohol drinking. The median NIHSS score at admission was 9 points. 104(17.16%) of patients underwent intravenous thrombolysis and 33(5.45%) underwent thrombectomy.9(1.49%) underwent intravenous thrombolysis add thrombectomy.194 (32.01%) patients developed an infection within first 7 days after admission.

### Evaluation of the prognostic value of NLR for PSI

**Fig. 1:**
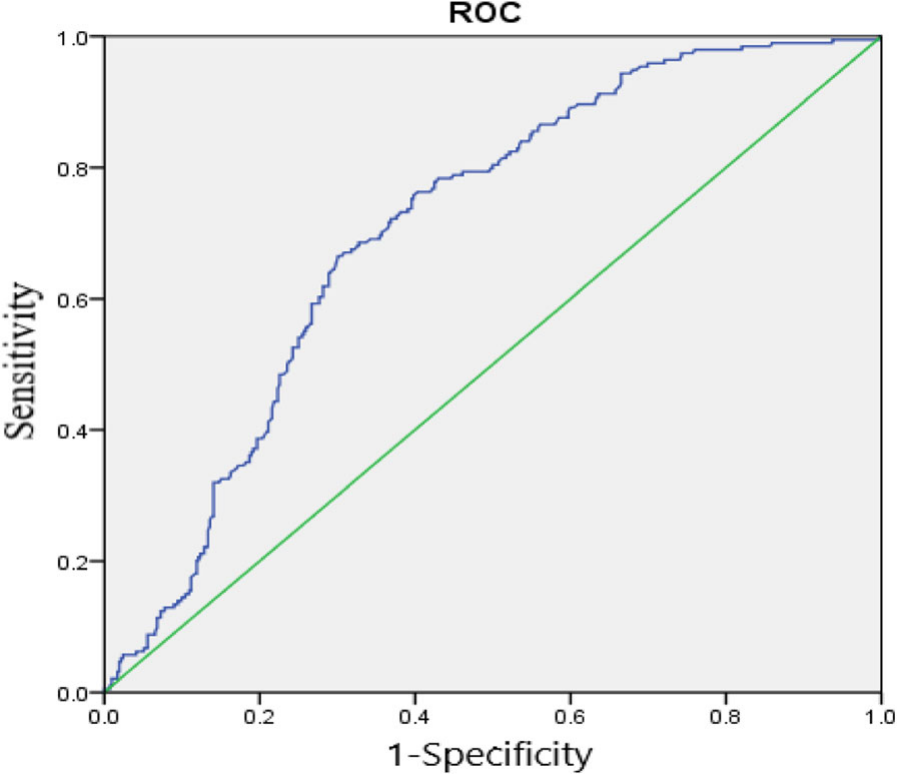
Receiver operating characteristic curve analysis for NLR for prognostic value for PSI

ROC curve analysis showed high accuracy for NLR to predict PSI with AUC of 0.72 (95% CI 0.68 to 0.76) (Fig. 1). Using a cut off value for NLR ≥ 5.79,the sensitivity was 65.98%, and the specificity was 70.63%, providing a positive predictive value (PPV) for PSI of 51.40%, and providing a negative predictive value (NPV) for PSI of 81.51%.

### Univariable models for predictors of PSI

194 (32.01%) patients had PSI with 7 days after AIS. One hundred twenty patients had SAP, 64 had UTI,10 patients had infections with other localization. Patients with NLR ≥ 5.79 experienced more SAP [33,73%(84/249) versus 10.08% (36/357), *P* < 0.001] and UIT[15.67% (39/249) versus 7.00% (25/357), *P* = 0.001]. Baseline characteristics of patients in the no PSI and PSI groups were compared (Table 1). At baseline, patients with PSI showed significantly older age (68.59 ± 10.72 vs 65.85 ± 10.06, P = 0.001), higher NIHSS [10(7–13) vs 8(6–11), *P* < 0.001],higher NLR[6.99(4.93–8.31) vs 4.25(2.92–6.45), *P* < 0.001], higher PCT(1.81 ± 0.86 vs 0.54 ± 4.92, *P* < 0.001), higher prevalence of indwelling urinary catheter (aOR1.83; 95%CI, 1.08–3.10; *P* = 0.026], and nasogastric tube feeding (aOR2.52; 95%CI, 1.38–4.59; *P* = 0.003) than patients with no PSI. Compared with patients no PSI, those with PSI had higher prevalence of NLR ≥ 5.79 (65.78% vs 29.37%, *P* < 0.001).

**Table 1:** Comparison of baseline characteristics between patients with no PSI and PSI groups

	no PSI group (412)	PSI group (194)	OR(95%CI)	P*
Age, y (Mean SD)	65.85 ± 10.06	68.59 ± 10.72		**0.001**
NIHSS score, median (IQR)	8(6–11)	10(7–13)		**< 0.001**
NLR, median (IQR)	4.25(2.92–6.45)	6.99(4.93–8.31)		**< 0.001**
Procalcitonin, μg/l (Mean SD)	0.54 ± 4.92	1.81 ± 0.86		**< 0.001**
NLR ≥ 5.79,n(%)	121(29.37)	128(65.78)	4.66(3.24–6.72)	**< 0.001**
Females,n(%)	220(53.40)	90(46.39)	0.73(0.52–1.03)	0.107
Men,n(%)	192(46.60)	104(53.61)	0.760.54–1.06)	0.107
BMI ≥ 24 kg/m, n(%)	117(28.40)	62(31.96)	0.79(0.56–1.13)	0.192
Hypertension,n(%)	273(66.26)	118(60.82)	0.79(0.56–1.13)	0.192
Current Smoking,n(%)	104(25.24)	59(30.41)	1.29 (0.89–1.89)	0.181
Current alcohol drinking,n(%)	122(29.61)	61(31.44)	1.09(0.75–1.58)	0.647
Diabetes, n(%)	138(33.50)	73(37.62)	1.20(0.84–1.71)	0.319
Hyperlipidemia,n(%)	237(57.52)	97(50.00)	0.74(0.52–1. 04)	0.082
Thrombolytic therapy, n(%)	68(16.50)	36(18.56)	1.15(0.74–1.80)	0.532
Thrombectomy, n(%)	25(6.07)	8(4.12)	0.67(0.30–1.50)	0.325
Thrombolytic therapy+ Thrombectomy,n(%)	5(1.21)	4(2.06)	1.71(0.46–6.45)	0.421
Family history of stroke,n(%)	84(20.39)	38(19.59)	0.95(0.62–1.50)	0.819
Etiological classification				
Large artery atherosclerosis,n(%)	137(33.25)	70(36.08)	1.13(0.79–1.62)	0.493
Cardioembolism,n(%)	69(16.75)	31(15.98)	0.95(0.60–1.50)	0.812
Lacunar, n(%)	122(29.61)	62(31.96)	1.12(0.77–1.61)	0.558
Other known causes, n(%)	5(1.21)	2(1.03)	0.85(0.16–4.41)	0.844
Undetermined, n(%)	82(19.90)	30(15.46)	0.74(0.47–1.16)	0.189
Nasogastric tube feeding	33(8.01)	39(20.10	2.89(1.75–4.76)	**< 0.001**
Indwelling urinary catheter	48(11.65)	52(26.80)	2.77(1.79–4.30)	**< 0.001**
Medications use				
Antiplatelet, n(%)	110(26.70)	57(29.38)	1.14(0.78–1.67)	0.491
Antihypertensive, n(%)	220(53.40)	99(51.03)	0.91(0.65–1.28)	0.586
Lipid-lowering medications, n(%)	182(44.17)	78(40.21)	0.85(0.60–1.20)	0.357

Bold indicates *P*-values less than 0.05.

*Comparison between no PSI and PSI groups. The data are presented as median values (interquartile range [IQR]), numbers (%), or mean values (±standard deviation). Categorical variables are expressed as frequency (percent) for *P* values Continuous variables are expressed as mean ± standard deviation (SD). Baseline characteristics were compared between the 2 subgroups by univariate analysis using Pearson χ2, distributions of continuous variables were determined by the Kolmogorov–Smirnov test, Mann–Whitney two sample test was applied in case of non-normal distributions

### Multivariable models on the association between NLR ≥ 5.79 and PSI

In unadjusted models, there was an association between NLR ≥ 5.79 and PSI group (OR,4.66;95%CI, 3.24–6.72, *P* < 0.001). In the multivariable logistic regression model after adjustment for age, baseline NIHSS score, sex, BMI, hypertension, current smoking, current alcohol drinking, diabetes, hyperlipidemia, thrombolytic therapy, thrombectomy, thrombolytic therapy+Thrombectomy, family history of stroke, etiological classification, nasogastric tube feeding, indwelling urinary catheter, and medications use, NLR ≥ 5.79 (aOR 4.52, 95% CI 3.02–6.76, *P* < 0.001), older age (aOR 1.03, 95% CI 1.00–1.05, *P* = 0.009), admission NIHSS (aOR 1.13, 95% CI 1.07–1.18, *P* < 0.001), indwelling urinary catheter (aOR 1.83, 95% CI 1.08–3.10, *p* = 0.026),and nasogastric tube feeding (aOR 2.52, 95% CI 1.38–4.59, *p* = 0.003) were independently associated with increased risk of PSI (Table 2).

**Table 2:** Multivariable Models Showing Predictors of PSI

	aOR (95% CI)	P*
NLR ≥ 5.79	4.52 (3.02–6.76)	**< 0.001**
Age	1.03(1.00–1.05)	**0.009**
Admission NIHSS	1.13(1.07–1.18)	**< 0.001**
Indwelling urinary catheter	1.83(1.08–3.10)	**0.026**
Nasogastric tube feeding	2.52(1.38–4.59)	**0.003**

Bold indicates *P*-values less than 0.05.

* Multivariable adjusted for age, baseline NIHSS score, sex, BMI, hypertension, current smoking, current alcohol drinking, diabetes, hyperlipidemia, thrombolytic therapy, thrombectomy, thrombolytic therapy+Thrombectomy, family history of stroke, etiological classification, nasogastric tube feeding, indwelling urinary catheter,and medications use

## Discussion

Stroke is the most common disabling and fatal disease in adults. More than 40% surviving stroke patients have neurological deficit symptoms and need to be cared for [24]. The factors that affect the prognosis of the patients are the severity of stroke and old age. In addition, infection has a negative effect on the outcome [22, 25, 26], which plays an important role in extending hospital stay, worsening of neurological outcome, developing more serious complications and death [26]. In this study, we found that 32.01% of patients had infection within 7 days after AIS, which was consistent with previous studies [27, 28].

NLR may reliably predict inflammatory response, which represents the information of innate immune system and adaptive immune system. NLR is easy to obtain in clinical practice, it is cost-effective. NLR increases with the progression of some diseases, especially in inflammatory diseases, and this increase is consistent with the development of some diseases. A large number of studies have shown that higher NLR might be an independent predictor of clinical outcome in a variety of diseases, including malignant tumors, acute respiratory distress syndrome, fibrotic liver disease and cardiovascular diseases [29–32]. At present, it has been paid more attention in the research of neurovascular diseases [33–35]. Many studies have found that baseline NLR levels are higher in some AIS patients, with the increase of neutrophils and the decrease of lymphocytes, higher NLR indicated the imbalanced interaction between central and peripheral inflammation after AIS [36, 37]. Many studies shown that higher NLR on admission increased the risk of poor prognosis in AIS patients [16–20], but its diagnostic value for predicting infections after AIS has not been extensively investigated. In this study, we aimed to investigate the association between increased NLR and PSI in patients with AIS, and we found that a higher NLR was associated with increased risk of PSI in patients with AIS. The PSI group had a significantly higher NLR than that of the non-PSI group.

There are several underlined mechanisms for the relationship between the NLR and PSI. One explanation might be that the interaction of the brain’s immune system is impaired after a stroke, leading to a stroke related immunosuppressive syndrome [25, 38]. Ischemic stroke initiates local inflammatory immune response in brain tissue. Inflammatory factor such as interleukin-6, tumor necrosis factor-α, interleukin-1, and matrix metalproteinase-9 release from endothelium and brain parenchyma, which can further potentiate tissue injury, which disrupt of the brain–blood barrier (BBB). Inflammatory factors can enter the systemic circulation through the broken BBB [39, 40]. Then, the systemic inflammatory response is changed through sympathetic pathway and hypothalamus pituitary adrenal axis [41], neutrophils are dedifferentiated and stimulated by growth factors [42], lymphocyte apoptosis, which increased the susceptibility of infection [43, 44]. Another explanation is that NLR is associated with stroke severity, and stroke severity is strong risk factor for infection [9, 45]. In our study, ROC curve analysis showed that NLR ≥ 5.79 was a useful marker for predicting infection. The AUC of 0.72 in PSI group further indicated that NLR was predictive of infection with high specificity, the result shown that the NLR ≥ 5.79 group had a significantly higher NIHSS than that of the NLR<5.79 group(9.73 ± 4.76 vs 8.94 ± 4.19, *P* = 0.038).

We observed that NLR ≥ 5.79 was strong associated with increased risk of PSI within 7 days. The percentage of PSI in the NLR ≥ 5.79 group was 51.41% (128/249), which was significantly higher than that in the NLR<5.79 group(18.49%,66/357) (*P* < 0.001). After adjusting for fully confounders, in addition to age, NIHSS, indwelling urinary catheter and nasogastric tube feeding, we found a significant association of NLR ≥ 5.79 with increased risk of PSI after AIS. These results suggested this association was independent of established risk factors, including age, baseline NIHSS score, sex, BMI, hypertension, current smoking, current alcohol drinking, diabetes, hyperlipidemia, thrombolytic therapy, thrombectomy, thrombolytic+thrombectomy therapy, family history of stroke, etiological classification, nasogastric tube feeding, indwelling urinary catheter, and medications use, and NLR detection could have prognostic value for infection among patients with AIS.

Some limitations of this study merit consideration. First, in this study, we relied on a single baseline NLR, we should monitor the changes of NLR dynamically, which might provide additional information on PSI. Second, due to incomplete data, we did not analyze the associated NLR with severity of infection, there also seemed to be an association between the NLR and infection severity. Third, we lack data on the possible influence of NLR on different infection (pneumonia, urinary tract infection, other location’s infection). Fourth, even if we exclude infected patients before admission, we cannot exclude the bias of undiagnosed infections that occurred before admission. However, despite these limitations, our research has the advantage that its large sample size, its analysis, including models adjusting for a wide variety of confounders.

## Conclusions

In conclusion, our study showed a positive correlation between increased NLR and PSI in patients with AIS. Early diagnosis of PSI is very important, which can reduce mortality and improve outcome. NLR may have potential predictive value in risk stratification of ischemic stroke. NLR can be obtained quickly and easily from blood cell count, which may help to select high-risk patients to start intervention in time, our results imply that this method might be applicable in AIS management.

## Data Availability

The datasets used and/or analyzed during the current study are available from the corresponding author on reasonable request.

## Abbreviations

CI: Confidence interval
M: Mean
OR: Odds ratio
SD: Standard deviation
NLR: Neutrophil-to-lymphocyte ratio
PSI: Post-stroke infection
SAP: Stroke-associated pneumonia
UTI: Urinary tract infection (UTI)
AIS: Acute ischemic stroke
IQR: Interquartile range
PCT: Procalcitonin
